# Biochemical Characterization of SARS-CoV-2 Spike RBD Mutations and Their Impact on ACE2 Receptor Binding

**DOI:** 10.3389/fmolb.2022.893843

**Published:** 2022-05-23

**Authors:** Abdullah Hoter, Hassan Y. Naim

**Affiliations:** ^1^ Department of Biochemistry, University of Veterinary Medicine Hannover, Hannover, Germany; ^2^ Department of Biochemistry and Chemistry of Nutrition, Faculty of Veterinary Medicine, Cairo University, Giza, Egypt

**Keywords:** SARS-CoV-2, spike, ACE2 interaction, RBD, double mutant, transmissibility

## Abstract

Infection of mammalian cells by SARS-CoV-2 coronavirus requires primary interaction between the receptor binding domain (RBD) of the viral spike protein and the host cell surface receptor angiotensin-converting enzyme 2 (ACE2) glycoprotein. Several mutations in the RBD of SARS-CoV-2 spike protein have been reported for several variants and resulted in wide spread of the COVID pandemic. For instance, the double mutations L452R and E484Q present in the Indian B.1.617 variant have been suggested to cause evasion of the host immune response. The common RBD mutations N501Y and E484K were found to enhance the interaction with the ACE2 receptor. In the current study, we analyzed the biosynthesis and secretion of the RBD double mutants L452R and E484Q in comparison to the wild-type RBD and the individual mutations N501 and E484K in mammalian cells. Moreover, we evaluated the interaction of these variants with ACE2 by means of expression of the S protein and co-immunoprecipitation with ACE2. Our results revealed that the double RBD mutations L452R and E484Q resulted in a higher expression level and secretion of spike S1 protein than other mutations. In addition, an increased interaction of these mutant forms with ACE2 in Calu3 cells was observed. Altogether, our findings highlight the impact of continuous S1 mutations on the pathogenicity of SARS-CoV-2 and provide further biochemical evidence for the dominance and high transmissibility of the double Indian mutations.

## 1 Introduction

The highly infectious coronavirus (severe acute respiratory syndrome coronavirus-2, SARS-CoV-2) emerged in 2019 and caused the most severe pandemic in the 21st century. Due to the high pathogenicity of SARS-CoV-2 and its rapid spread, more than 270 million people have been infected and over 5 million people died by coronavirus disease 2019 (COVID-19) (WHO, accessed on 20 December 2021). Fortunately, the speed of developing efficient vaccines and the extensive vaccination campaign constitute excellent assets in combating further transmission of the pandemic (Forni et al., 2021; Mahase, 2021). On the other hand, emerging mutations within the viral genome can change the pathogenic potential of the virus and enable evasion of subsequent immune response (Garcia-Beltran et al., 2021; Greaney et al., 2021; Harvey et al., 2021). Therefore, it is most likely that those adaptive mutations might hinder the vaccination efforts and limit the efficacy of certain SARS-CoV-2 vaccines (Ferreira et al., 2021; Jia and Gong, 2021). Clear recent evidence on this subject is what we currently witness from the SARS-CoV-2 variant Omicron that has been reported to significantly escape the existing COVID-19 vaccines (Zhang et al., 2021; Chen et al., 2022).

Cell entry into host cells is a crucial step in the SARS-CoV-2 infection and pathogenesis that represent an attractive hub for many preventive measures and vaccination strategies (Li, 2016; Du et al., 2017; Shang et al., 2020a). SARS-CoV-2 binds initially to angiotensin-converting enzyme 2 (ACE2) on the host cell surface *via* its spike (S) protein before it undergoes endocytosis and fusion with the lysosomal membrane (Shang et al., 2020a; Hoffmann et al., 2020). SARS-CoV-2 S protein exists as a homotrimer glycoprotein on the virus envelope where each S monomer comprises an S1 subunit, which interacts with ACE2 through the receptor binding domain (RBD), and S2 subunit, which mediates the viral membrane fusion with the host cell membrane (Li, 2016; Duan et al., 2020; Lan et al., 2020; Mittal et al., 2020).

In host cells, the SARS-CoV-2 S glycoprotein is primarily synthesized on the endoplasmic reticulum (ER) as a precursor protein composed of 1,273 amino acids with its N-terminal signal sequence peptide (Duan et al., 2020; Liu et al., 2020). Following the synthesis and N-glycosylation in the ER, the S-glycoprotein monomers form a trimer protein that is trafficked to the Golgi complex, where O-linked glycans are added and modification of the high-mannose oligosaccharide side chains into complex forms occur (Zhang and Wang, 2016; Joshi et al., 2018; Andersen et al., 2020).

Throughout the pandemic, particular mutations in the S protein have emerged and were shown to increase the rate of transmission and/or infectivity of the virus (Korber et al., 2020; Volz et al., 2021). For instance, N501Y, which is located in the S1-RBD, increases the binding affinity to ACE2 (Barton et al., 2021; Upadhyay et al., 2021). E484K mutation coexists with other mutations such as K417N/T and N501Y in variants of concern (VOCs) such as B.1.351 and P.1, respectively (Collier et al., 2021; Ferreira et al., 2021). The two mutations L452R and E484Q, characterizing the variant B.1.617 that arose in India, have been reported to reduce the antibody response toward newly generated mRNA vaccines (Ferreira et al., 2021). L452R mutation has been shown to increase the viral load and enhance the virus transmissibility (Ferreira et al., 2021). Additionally, the data obtained with pseudotyped virus (PV) particles manifested that L452R caused 3–6 times reduction in sensitivity to neutralizing antibodies (Motozono et al., 2021). E484Q mutation, which simultaneously exists with L452R in the S1-RBD, has been associated with increased ACE2 binding and immune escape (Cherian et al., 2021).

In the present study, we analyzed the biosynthetic forms and glycosylation of intracellular and secreted forms of key S1-RBD mutations that have been observed in VOCs such as the double mutant E484Q and L452R, E484K, and N501Y. Moreover, we developed an *in vitro* system to biochemically evaluate the interaction potential of those mutations with ACE2 in mammalian cells. Our results showed that the double mutants L452R and E484Q were comparatively highly secreted and interacted strongly with ACE2 in the human lung Calu3 cells.

## 2 Materials and Methods

### 2.1 Chemicals and Reagents

Cell culture dishes were obtained from Sarstedt (Nümbrecht, Germany). Cell culture reagents including Dulbecco’s modified Eagle medium (DMEM), streptomycin, penicillin, fetal calf serum (FCS), and trypsin-EDTA were purchased from Sigma-Aldrich (Deisenhofen, Germany). Protein A Sepharose, protease inhibitors, DEAE-dextran, n-dodecyl β-D-maltoside, and Triton X-100 (TX-100) were bought from Sigma-Aldrich (Deisenhofen, Germany). Western blot reagents including Rotiphorese® Gel 30 (37.5:1) acrylamide, sodium dodecyl sulfate (SDS), N,N,N′, N′-tetramethylenediamine (TEMED), dithiothreitol (DTT), and polyvinylidene fluoride (PVDF) membrane were purchased from Carl Roth GmbH (Karlsruhe, Germany). Tris and paraformaldehyde were obtained from Carl Roth GmbH (Karlsruhe, Germany) as well. Endo-β-N-acetylglucosaminidase H (Endo H) was bought from Sigma-Aldrich, Darmstadt, Germany. All the reagents were of analytical grade.

### 2.2 Immunological Reagents

Rabbit polyclonal antibody (PAb) anti-SARS-CoV-2 (2019-nCoV) spike was obtained from Sino Biological. Horseradish peroxidase-conjugated protein A (protein A-HRP) was purchased from Cell Signaling Technology (cat. #12291S), and mouse anti-FLAG was from Sigma-Aldrich Chemie GmbH (Munich, Germany). Anti-β-actin (C4) was bought from Santa Cruz Biotechnology, Inc. (Heidelberg, Germany). Goat anti-rabbit Alexa Fluor 568 and goat anti-mouse Alexa Fluor dye 488 were obtained from Invitrogen (Karlsruhe, Germany).

### 2.3 Cell Culture

COS-1 cells (African green monkey kidney fibroblast-like cell line) were cultivated in Dulbecco’s modified Eagle medium containing 1 g/L glucose (low glucose) and supplemented with 10% fetal bovine serum, 100 U/ml of penicillin, and 0.1 mg/ml of streptomycin (PAN-Biotech). Calu3 cells (human lung cancer cell line) were cultured in DMEM containing 4.5 g/L glucose (high glucose) and supplemented with 10% fetal bovine serum (Biochrom), 2 mM glutamine, 100 units/ml penicillin, and 0.1 mg/ml streptomycin. Both cell lines were incubated at 37°C and 5% CO_2_ in a humidified atmosphere.

### 2.4 Expression Plasmids and Generation of S1-RBD Mutations

pCG1 expression vector harboring the sequence encoding S1 subunit of SARS-S1 protein fused to the Fc portion of human immunoglobulin was kindly provided by Dr. Markus Hoffmann (Hoffmann et al., 2020) and used as a template for the generation of different S1 mutations. Point mutations of S1 protein were generated using the mutagenesis primers listed in Table 1. Amplification was performed using Phusion DNA polymerase (Thermo Fisher, Schwerte, Germany).

**TABLE 1:** List of mutagenesis primers used to generate S1 mutations of interest.

Primer	Sequence (5’-> 3′)
S1-L452R-For	AAT​TAC​CGG​TAC​CGG​CTG​TTC​CG
S1-L452R-Rev	TAC​CGG​TAA​TTG​TAG​TTG​CCG​CCG
S1-E484Q-For	TGC​AAG​GCT​TCA​ACT​GCT​ACT​TCC​C
S1-E484Q-Rev	AGT​TGA​AGC​CTT​GCA​CGC​CGT​TAC
S1-E484K-For	TGA​AAG​GCT​TCA​ACT​GCT​ACT​TCC​C
S1-E484K-Rev	AGT​TGA​AGC​CTT​TCA​CGC​CGT​TAC
S1-N501Y-For	ACA​TAT​GGC​GTG​GGC​TAT​CAG​C
S1-N501Y-Rev	ACG​CCA​TAT​GTG​GGC​TGA​AAG​C

All mutagenized plasmids were further sequenced (Macrogen, Amsterdam, Netherlands) to confirm the generated mutations. FLAG-tagged camel endoplasmin or GRP94 (pCMV-cGRP94-FLAG) that shares 100% homology with human GRP94 was constructed, as described previously (Hoter et al., 2018).

### 2.5 Transfection, Cell Lysis, Immunoprecipitation, and Glycosylation

COS-1 cells were transfected with plasmids encoding either wild-type S1 protein (S1-Wt) or S1 mutations including S1-E484K, S1-N501Y, or the double mutation (E484Q and L452) using the diethylaminoethyl (DEAE)-dextran method, as described previously (Naim et al., 1991). Co-expression experiments of S1 plasmids and cGRP94 were carried out by transfecting two plasmids using the same method. Twenty four hours after transfection, media of transfected COS-1 cells were replaced by fresh DMEM and kept for 8 h before they were finally replaced with FCS-free DMEM and kept overnight. Then 48 h after transfection, the cells were lysed using 1% TX-100 or 10 mM n-dodecyl β-D-maltoside in phosphate-buffered saline (PBS), pH 8.0, and a mixture of protease inhibitors. Post-nuclear lysates were subjected to Western blotting or first mixed with protein A Sepharose (PAS) alone to immunoprecipitate S1-Fc protein or bound to mouse anti-FLAG antibody to pull down cGRP94. For glycosylation experiments, the immunoprecipitates were treated or not treated with Endo H, as previously described (Naim et al., 1991).

### 2.6 Assessment of S1 Interaction With ACE2 in Calu3 Cells

Two days after transfection, the FCS-free media of COS-1 cells were collected and centrifuged at 1000 rpm for 2 min to remove cellular debris and dead cells. Debris-free media were collected and added to post-confluent Calu-3 cells that have been washed 2 times with PBS. Binding of secretory S1 to Calu3 ACE2 was allowed to occur for 2 hours at 4°C to inhibit endocytosis. After 2 h, both COS-1 media and Calu3 cellular extracts were subjected to immunoprecipitation, as described in the previous section.

### 2.7 SDS–Polyacrylamide Gel Electrophoresis and Western Blotting

All immunoprecipitates were washed 3 times with PBS and boiled in Laemmli buffer containing 100 μM DTT for 5 min at 95°C before their resolving on 8% SDS gels. Cellular lysates of the respective experiments were measured for protein concentration by using the Bradford method (Bradford, 1976) before their separation on SDS gels. Resolved proteins were transferred to a PVDF membrane and then blocked in 5% non-fat dry milk in PBST buffer (PBS containing 0.1% Tween-20) for 1 h at room temperature. Each protein was detected using its specific antibody: rabbit PAb anti-spike or protein A-HRP for detection of S1, mouse anti-FLAG for detection of cGRP94, and mouse anti-β-actin for detection of β-actin. The antibody–antigen complex was detected with the HRP secondary antibody (Thermo Fisher Scientific, Schwerte, Germany) and visualized via a ChemiDoc MP™ Touch Imaging System (Bio-Rad, Munich, Germany).

### 2.7 Confocal Fluorescence Microscopy

COS-1 cells were seeded on coverslips and transfected with the respective plasmids, as described before (Hoter and Naim, 2021). Briefly, the primary antibodies used were rabbit PAb anti-spike and mouse anti-FLAG, while the secondary antibodies used were rabbit IgG antibody carrying Alexa Fluor 568 and anti-mouse IgG antibody carrying Alexa Fluor 488. Visualization of proteins was performed by using a Leica TCS SP5 confocal microscope with an HCPL APO 63 ×1.3 glycerol immersion objective.

### 2.8 Modeling

To characterize S protein mutations, the crystal structure of SARS-CoV-2 S glycoprotein in complex with ACE2 was retrieved from the Protein Data Bank (PDB ID: 6m0j (Lan et al., 2020)). The mutations in the RBD of S protein were mapped using Chimera software (Pettersen et al., 2004).

### 2.9 Statistical Analyses

Protein bands were quantified using Image Lab software (Bio-Rad Laboratories GmbH). The calculations were analyzed using Microsoft Excel and represented using Prism 5 software. Statistical analyses include paired or unpaired Student’s t-test, and the presented error bars indicate the standard error of the mean (SEM). The results shown are representative of at least three independent repeats, and significance is designated as *p < 0.05, **p < 0.005, ***p < 0.0005, and ****p < 0.00005.

## 3 Results

### 3.1 Characterization of SARS-CoV-2 Spike S1 Subunit and Its Variants in COS-1 Cells

**FIGURE 1:**
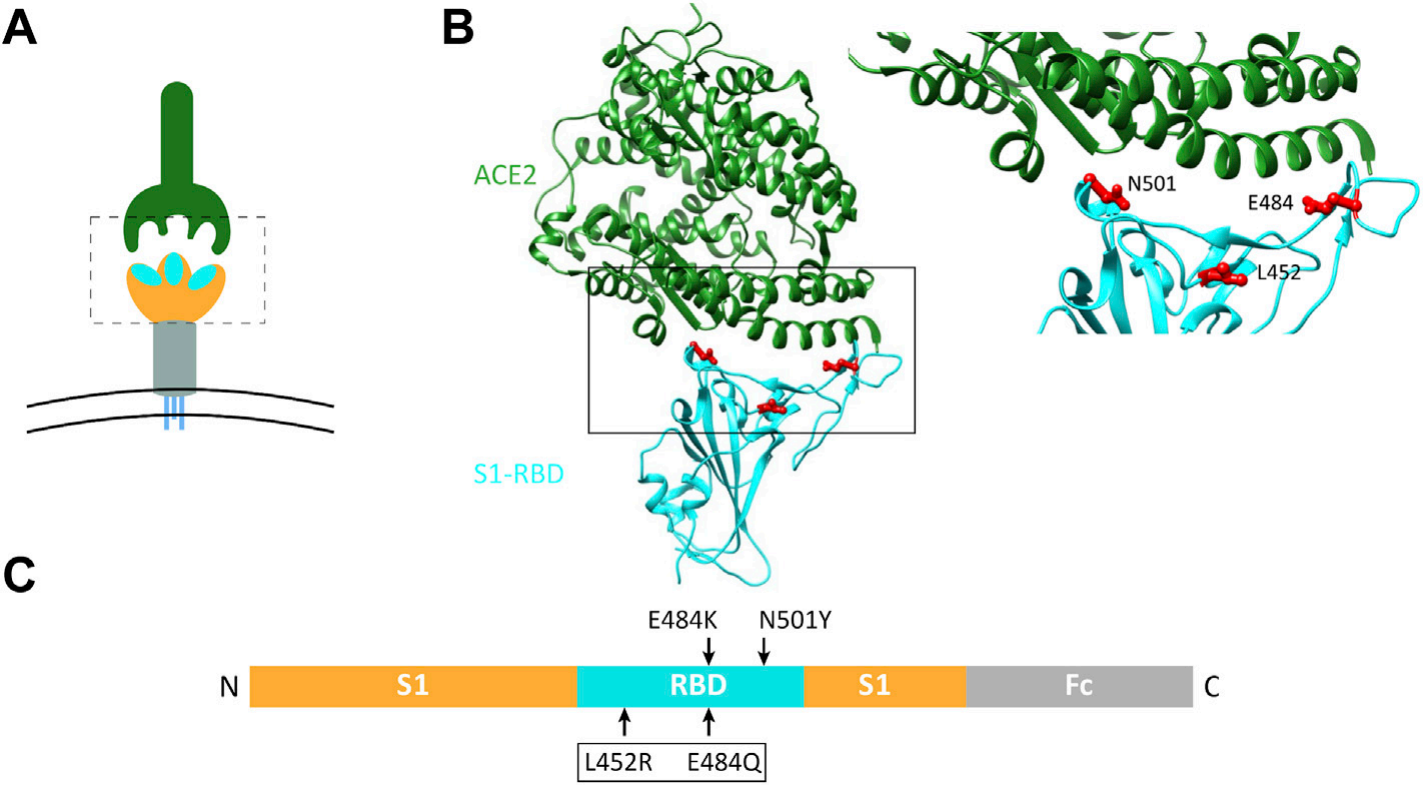
Schematic representation of the host ACE2 receptor and spike RBD variants analyzed in this study. **(A)** Three-dimensional (3D) representation of coronavirus spike protein (lower graphic) and the host ACE2 receptor (upper graphic). Spike protein forms a homotrimer with three S1-RBD heads (enclosed in dotted box). **(B)** Structural model of SARS-CoV-2 RBD (cyan) interacting with human ACE2 (dark green), based on PDB 6m0j (Lan et al., 2020). The area bordered by the box is magnified on the right to show the sites of mutations analyzed in the current study. **(C)** Linear representation of the Fc-tagged S1 cDNA plasmid used. Arrows indicate sites of mutated amino acids.

Since the double mutations L452R and E484Q of S1 (dMu) have been associated with the immune evasion and the high infectivity rates reported in late 2020 and early 2021 in India (Ferreira et al., 2021), we chose this combined mutant to analyze its biochemical behavior in comparison with the wild-type S1 or other single S1 mutations including E484K and N501Y, which occurred in different common lineages. For instance, the E484K mutation has appeared in the Beta (B.1.351), Kappa (B.1.617.1), Gamma (P.1), and P.2, while the N501Y mutation has been found in the Alpha (B.1.1.7), Beta, and Gamma variants (Harvey et al., 2021). Notably, all these mutations are located in the ACE2-binding interface of the S1-RBD, as represented in Figure 1.

**FIGURE 2:**
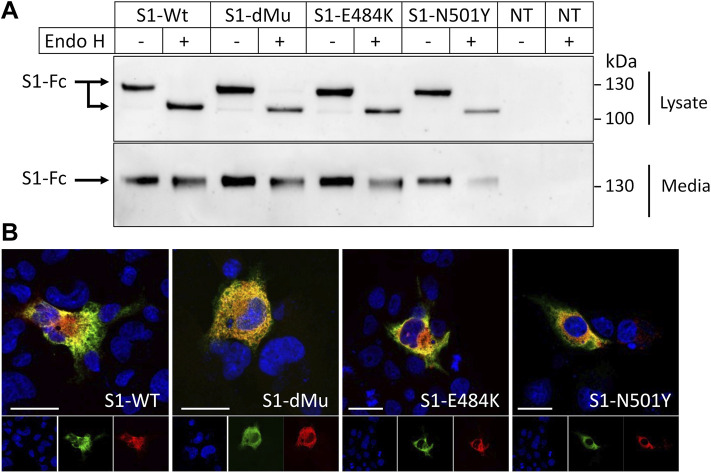
Characterization of subcellular and secretory forms of spike S1 proteins. **(A)** COS-1 cells were transiently transfected or non-transfected (NT) with plasmids encoding Wt-S1, dMu (L452 and E484), E484K, and N501Y S1 mutations. After 48 h of transfection, proteins were immunoprecipitated from both cell lysates, and media were then treated or not treated with Endo H. Cellular and secretory S1 proteins were immunoprecipitated and analyzed by Western blot. The upper panel shows a 130-kDa protein that shifted to a lower molecular weight following Endo H treatment. **(B)** Subcellular localization of S1 variants in the transiently transfected COS-1 cells. S1-Wt and mutant proteins were expressed in the COS-1 cells grown on coverslips, and their subcellular localization in the ER was investigated by immunofluorescence and visualized by confocal microscopy. The cGRP94 plasmid that encodes the ER chaperone GRP94 was used as an ER marker. Scale bars, 10 and 20 μm.

To characterize the S1 subunit of spike protein and variants of interest, we transfected their encoding plasmids and analyzed their expressed proteins in COS-1 cells as a model of mammalian cells. These cells have been widely used as an established system to study protein trafficking and posttranslational modifications of many viral glycoproteins (Doedens et al., 1997; Elliott and O’Hare, 1997). Figure 2A shows that immunoprecipitation of the media revealed the mature Endo H-resistant complex glycosylated forms of the Wt-S1 and dMu, E484K, and N501Y mutant counterparts. The detergent cellular extracts, by contrast, contained exclusively the Endo H-sensitive 130-kDa mannose-rich polypeptide at steady state compatible with a rapid secretion of the Wt-S1 and the S1 mutants after complex glycosylation in the Golgi.

These data were confirmed by immunofluorescence analyses (Figure 2B), which revealed an exclusive intracellular localization of the Wt-S1 and the mutants in the ER as assessed by their co-localization with the ER chaperone GRP94.

### 3.2 The Secretion of the dMu S1 Variant Is Substantially Increased as Compared to Wt-S1 From COS-1 Cells

**FIGURE 3:**
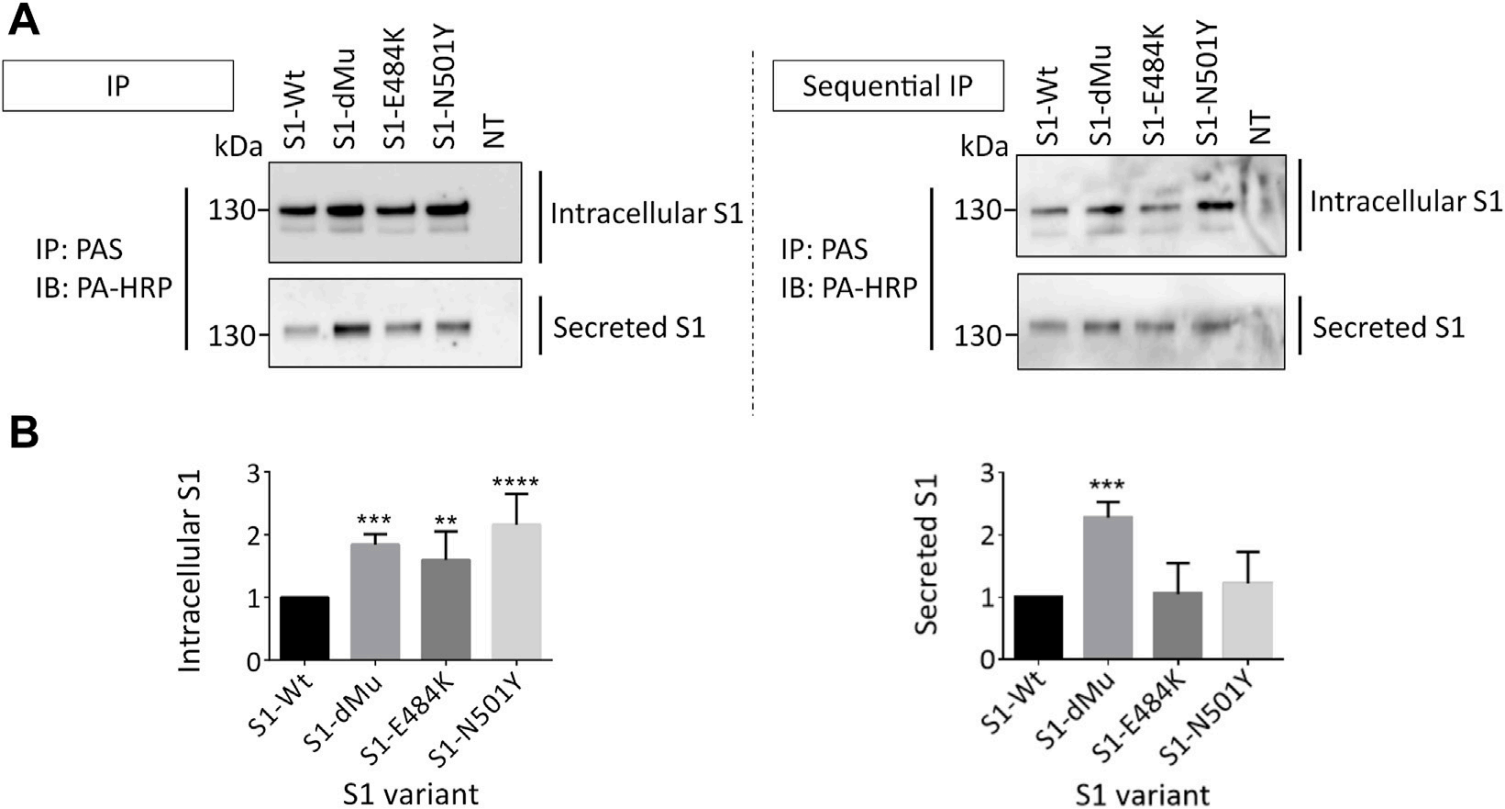
dMu, E484K, and N501Y variants of S1 are comparatively highly expressed and secreted in COS-1 cells. **(A)** Transfected COS-1 cells were lysed 48 h after transfection. Equal protein-containing lysates (upper left blot) and collected media (lower left blot) of each S1 mutant were pulled down using protein A-bound Sepharose (PAS) and analyzed using SDS-PAGE on 8% slab gels. To confirm these expression profiles, we carried out parallel sequential immunoprecipitation of both lysates (upper right blot) and media (lower right blot), which reflected higher intracellular and secretory protein levels in the case of S1-dMu and S1-N501Y mutants. **(B)** Comparison between relative expression levels of S1 variants in COS-1 cells (left bar graph) and their secretory proteins collected from the media (right bar graph) (*n* = 4). Data are normalized to the wild type and expressed as arbitrary units.

We next examined the effects elicited by the mutations on the intracellular synthetic levels of the S1 protein relative to Wt-S1. For this, immunoprecipitation of the S1 proteins from equal amounts of cellular lysates was performed and the protein levels compared to actin as a housekeeping protein. As shown in Figure 3 (left panel), all the S1 mutants including dMu (L452R and E484Q), E484K, and N501Y revealed higher subcellular and secretory levels than Wt-S1 and E484K. Markedly, an increase of 2.5-fold in the secretion rate of the dMu mutant was observed as compared to Wt-S1 and the mutants S1-E484K and S1-N501Y. To further substantiate this finding, we performed sequential immunoprecipitation on cellular lysates and the media of the variants and obtained similar results (Figure 3, right panel). Direct analysis of the intracellular levels of the S1 protein in cellular lysates of the transfected COS-1 cells revealed low levels due to the rapid secretion of the S1 protein into the media.

### 3.3 Interaction of S1 Variants With ACE-2 in Calu3 Cells Reveal Strong ACE2 Interaction With S1-dMu Mutant

**FIGURE 4:**
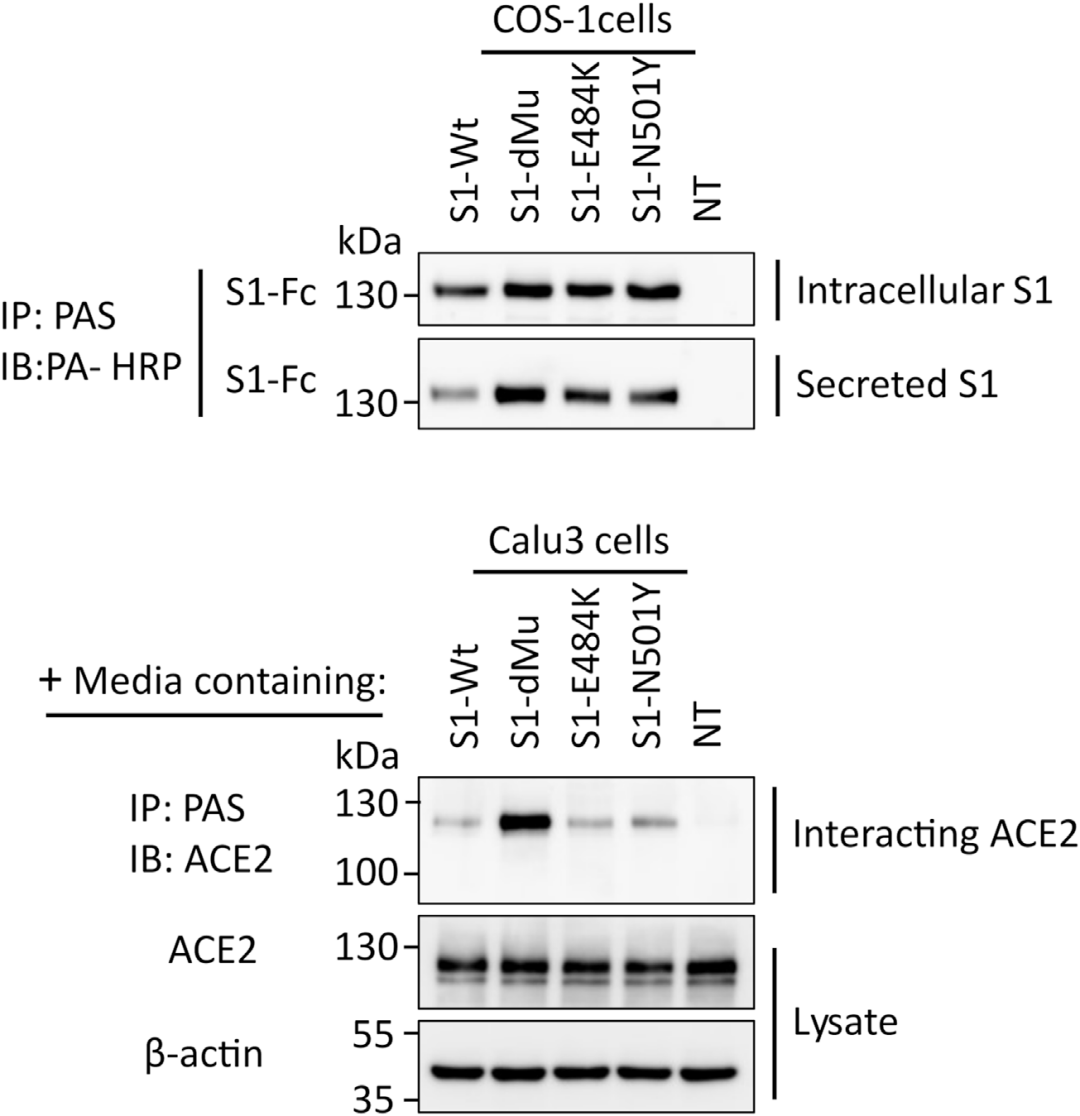
S1-dMu mutant interacts avidly with the ACE2 receptor in Calu3 cells. After 48 h of transfection of COS-1 cells, S1 variants were analyzed for their expression and secretion, as explained before (upper two blots). Equal amounts of COS-1 media containing secretory S1 proteins were allowed to bind to the ACE2 receptor expressed in Calu3 cells for 2 h at 4°C. Following the time specified for interaction, Calu3 cells were washed twice with PBS, pH 7.4 and lysed, as described before. Cellular lysates were analyzed for ACE2 expression where actin was used as housekeeping control protein (lowest two blots). To assess the interacting ACE2, the remaining major part of Calu3 lysate was subjected to immunoprecipitation using pAb anti-ACE2 and blotted against ACE2. Notably, the S1-dMu mutant showed stronger interaction capability with ACE2 than the other S1 proteins as revealed in the upper blot of the Calu3 cells.

To study the interaction of the S1 protein with its receptor ACE2, equal amounts of the media containing the S1 protein isoforms secreted from the transfected COS-1 cells were added to human lung Calu3 cells. This binding assay was performed at 4°C for 2 h, thus excluding any possible ligand–receptor endocytosis. We first assessed the expression levels of the S1 proteins in the media that have been utilized in the binding assays. Figure 4 shows similar variations in the expression levels of the S1 mutants and wild type, thus confirming the data obtained in Figure 3. We also assessed the expression levels of ACE2 in Calu3 cells subjected to the binding studies, and here again, the results revealed similar intensities of the ACE2 protein bands in the lysates in all experimental samples. The amount of ACE2 protein in the co-immunoprecipitates was next analyzed, which demonstrated variations in the binding capacities of ACE2 to S1 depending on the mutation. While there were minor variations between the ACE2 protein band bound to S1-Wt and to the S1-N501Y and S1-E484K mutants, the binding capacity of S1-dMu to ACE2 was several orders of magnitude higher than that of the other examined samples.

## 4 Discussion

Increased transmissibility and infectivity of SARS-CoV-2 are a global health concern. The emerging mutations in the spike protein of SARS-CoV-2, particularly those found in the RBD, represent an interesting area of investigation because they can directly affect the viral entry to host cells *via* modulating ACE2 binding (Shang et al., 2020a; Tian et al., 2021). In the present study, we analyzed key mutations in the S1 domain of the S protein including N501Y, E484K, and dMu (L452R, E484Q) that emerged and were observed in various lineages known by their high spread and infectivity. Overall, the secretion of the S1 protein, whether wild type or mutants, was extremely rapid after maturation in the Golgi and acquisition of an Endo H-resistant form, since only the mannose-rich immature form of this protein was revealed in the cellular lysates and the mature Endo H-resistant form in the cell culture medium. The higher expression levels of the analyzed S1 mutants than the wild-type S1, particularly the mutant containing the L452R and E484Q mutations, dMu, likely reflect an increased spike intensity and/or stability (Zhang et al., 2020; Upadhyay et al., 2021). Recent studies on RBD mutations showed variation in the expression levels of RBD mutations expressed in the HEK293 cells, although thermal stabilities of several RBD mutants were not affected when compared with the wild-type RBD (Upadhyay et al., 2021).

Notably, the relatively increased secretion of the S1-dMu in the media also correlates with the increased intracellular levels. This, in turn, resulted in an enhanced binding to ACE2 in the Calu3 cells as revealed by the co-interaction assay. These results provide clear biochemical evidence for the high transmissibility and infectivity of the dMu in India and raise the questions like to which extent those mutations can be advantageous to the virus (Barton et al., 2021; Cherian et al., 2021; Ferreira et al., 2021). On the other hand, it is not excluded as those dMu in S1-RBD modulate the affinity of binding between RBD and ACE2 (Barton et al., 2021). Previous studies on enveloped viruses, including SARS-CoV-2, showed the ability of those viruses to acquire improved ranges of affinities between the viral membrane glycoproteins and host cell receptors by means of their frequently occurring mutations. Consequently, increased affinities might lead to more efficient binding to host cells and cell entry (Hasegawa et al., 2007). For instance, in influenza virus, the estimated affinity is in the millimolar range, while in case of human immunodeficiency virus (HIV), the affinity to host receptors reached the nanomolar range (Takemoto et al., 1996; Ugolini et al., 1999; Skehel and Wiley, 2000; Hasegawa et al., 2007). Recent studies showed that S1-RBD binds to ACE2, with an affinity of 6–133 nM (Shang et al., 2020b; Wu et al., 2020; Zhang et al., 2020; Laffeber et al., 2021; Liu H. et al., 2021), and common S mutations including N501Y, E484K, and S477N could enhance the binding affinity to ACE2 and improve virus transmissibility (Barton et al., 2021). Notably, the N501Y present in the Alpha variant positively impacted the transmission rate of SARS-CoV-2 (Liu Y. et al., 2022). Here, we show that the dMu modification of S1-RBD leads to an increased secretion and also binding affinity to ACE2.

Altogether, the results add to the current knowledge on the impact of viral mutations in S1 on the interaction of SARS-CoV-2 with the ACE2 receptor and the potential role in its spreading and infectivity. Moreover, the implemented immunoprecipitation/co-immunoprecipitation under *in vitro* native conditions provides a solid biochemical approach to study the secretion and interaction of S1 with the ACE2 receptor. Our data demonstrated variations in the interactions rendering this methodology suitable to be applied to other variants of interest such as Omicron, Delta, Beta, and newly emerging variants like XD, XE, and XF. Together with other advanced biophysical techniques used for such screenings such as plasmon resonance (SPR) and bilayer interferometry (BLI) (Barton et al., 2021), an overall understanding of the impact of these variants on the mechanism of interaction of S with ACE can be achieved.

## Data Availability

The raw data supporting the conclusion of this article will be made available by the authors, without undue reservation.
